# Nutritional status at age 1 year in patients born with esophageal atresia: A population-based, prospective cohort study

**DOI:** 10.3389/fped.2022.969617

**Published:** 2022-08-04

**Authors:** Suzanne Depoortere, Alexandre Lapillonne, Rony Sfeir, Arnaud Bonnard, Thomas Gelas, Nicoleta Panait, Pierre-Yves Rabattu, Audrey Guignot, Thierry Lamireau, Sabine Irtan, Edouard Habonimana, Anne Breton, Virginie Fouquet, Hossein Allal, Frédéric Elbaz, Isabelle Talon, Aline Ranke, Michel Abely, Jean-Luc Michel, Joséphine Lirussi Borgnon, Philippe Buisson, Françoise Schmitt, Hubert Lardy, Thierry Petit, Yann Chaussy, Corinne Borderon, Guillaume Levard, Clara Cremillieux, Cécilia Tolg, Jean Breaud, Olivier Jaby, Céline Grossos, Philine De Vries, Myriam Arnould, Cécile Pelatan, Stephan Geiss, Christophe Laplace, Maéva Kyheng, Audrey Nicolas, Madeleine Aumar, Frédéric Gottrand

**Affiliations:** ^1^Univ. Lille, CRACMO Reference Center for Rare Esophageal Diseases, CHU Lille, INFINITE U1286, Lille, France; ^2^University Hospital APHP Necker-Enfants Malades, Paris, France; ^3^University Hospital APHP Robert Debré, Paris, France; ^4^University Hospital of Lyon, Lyon, France; ^5^University Hospital of Marseille, Marseille, France; ^6^University Hospital of Grenoble Alpes, Grenoble, France; ^7^University Hospital of Nantes, Nantes, France; ^8^University Hospital of Bordeaux, Bordeaux, France; ^9^University Hospital APHP Armand Trousseau, Paris, France; ^10^University Hospital of Rennes, Rennes, France; ^11^University Hospital of Toulouse, Toulouse, France; ^12^University Hospital APHP Kremlin Bicêtre, Paris, France; ^13^University Hospital of Montpellier, Montpellier, France; ^14^University Hospital of Rouen, Rouen, France; ^15^University Hospital of Strasbourg, Strasbourg, France; ^16^University Hospital of Nancy, Nancy, France; ^17^University Hospital of Reims, Reims, Champagne-Ardenne, France; ^18^University Hospital of La Réunion, Saint Denis de la Réunion, France; ^19^University Hospital of Dijon, Dijon, France; ^20^University Hospital of Amiens, Amiens, France; ^21^University Hospital of Angers, Angers, France; ^22^University Hospital of Tours, Tours, France; ^23^University Hospital of Caen, Caen, France; ^24^University Hospital of Besançon, Besançon, France; ^25^University Hospital of Clermont-Ferrand, Clermont-Ferrand, France; ^26^University Hospital of Poitiers, Poitiers, France; ^27^University Hospital of Saint-Etienne, Saint-Etienne, France; ^28^University Hospital of Fort De France, Martinique, Fort de France, France; ^29^University Hospital of Nice, Nice, France; ^30^University Hospital of Créteil, Créteil, France; ^31^University Hospital of Limoges, Limoges, France; ^32^University Hospital of Brest, Brest, France; ^33^General Hospital of Orléans, Orléans, France; ^34^General Hospital of Le Mans, Le Mans, France; ^35^General Hospital of Colmar, Colmar, France; ^36^University Hospital of Point à Pitre, Guadeloupe, Point à Pitre, France; ^37^CHU Lille–Department of Biostatistics, Lille, France

**Keywords:** undernutrition, stunting, catch-up, growth, prematurity, small for gestational age, syndromic

## Abstract

**Objective:**

Despite recent progress in caring for patients born with esophageal atresia (EA), undernutrition and stunting remain common. Our study objective was to assess nutritional status in the first year after birth with EA and to identify factors associated with growth failure.

**Study design:**

We conducted a population-based study of all infants born in France with EA between 2010 and 2016. Through the national EA register, we collected prenatal to 1 year follow-up data. We used body mass index and length-for-age ratio Z scores to define patients who were undernourished and stunted, respectively. Factors with *P* < 0.20 in univariate analyses were retained in a logistic regression model.

**Results:**

Among 1,154 patients born with EA, body mass index and length-for-age ratio Z scores at 1 year were available for about 61%. Among these, 15.2% were undernourished and 19% were stunted at the age of 1 year. There was no significant catch-up between ages 6 months and 1 year. Patients born preterm (41%), small for gestational age (17%), or with associated abnormalities (55%) were at higher risk of undernutrition and stunting at age 1 year (*P* < 0.05). Neither EA type nor surgical treatment was associated with growth failure.

**Conclusion:**

Undernutrition and stunting are common during the first year after birth in patients born with EA. These outcomes are significantly influenced by early factors, regardless of EA type or surgical management. Identifying high-risk patient groups with EA (i.e., those born preterm, small for gestational age, and/or with associated abnormalities) may guide early nutritional support strategies.

## Introduction

Esophageal atresia (EA), with or without tracheoesophageal fistula (TEF), is a rare congenital disorder that occurs in 1.9 per 10,000 births in France (1). This condition makes oral feeding impossible and without surgical treatment, exposes the infant to inhalation of food, saliva, and gastric fluid.

In recent decades, thanks to medical and surgical care improvements, survival rates have increased to 95% (1). Although more patients reach adulthood, they remain exposed to multiple complications during infancy (2–4), including surgical (anastomosis leakage, TEF recurrence, anastomotic stricture), digestive (gastroesophageal reflux disease [GERD], esophageal dyskinesia, dumping syndrome, eosinophilic esophagitis, Barrett's esophagus), and respiratory (tracheomalacia, bronchopneumopathy), as well as complications from possible underlying conditions. These can cumulatively impair growth by reducing food intake (*via* dysphagia, vomiting, oral aversion, food blockages, or inhalation) and increasing energy expenditure (from dyspnea, inflammation, or frequent infections).

Previous retrospective (5–9) and monocentric (5–10) studies have shown a high risk of early-life undernutrition or stunting in patients born with EA. Identified risk factors include low birth weight (10), low weight at discharge (9), GERD (7), anti-reflux surgery (10), and needing a second surgery in the first year after birth (11).

Preliminary analyses of the first two registry years showed that 15% of patients were underweight [Z score weight/age ≤ 2 standard deviations (SDs)] at the age of 1 year (5). Herein, we evaluated nutritional status at ages 6 months and 1 year among a population-based cohort of patients born with EA. Secondary objectives were to examine growth dynamics (i.e., catch-up) from 6 months to 1 year and to identify risk factors for stunting and undernutrition at the age of 1 year.

## Materials and methods

Data were from the French EA register, created in 2008. This population-based prospective epidemiological register (1) uses two forms to collect data on every patient born with EA in France. The first form is filled in during the initial hospitalization, the second is completed at the end of the first year of usual follow-up. Both forms were validated by a multidisciplinary committee of national experts, including epidemiologists, obstetricians, neonatologists, surgeons, and pediatricians (12) from 37 centers performing neonatal surgery in France and overseas.

Herein, we included all patients born with EA in France between January 1, 2010 and December 31, 2016. We extracted the following data: antenatal ultrasound suspicion of EA; pregnancy type (singleton, twins, multiples); gestational age at birth; sex; anthropometry at birth; type of EA according to Ladd classification (13); associated abnormalities and types; syndromic associations (14, 15); surgery type (esophageal anastomosis with or without lengthening artifice, colic transposition or gastric transposition); anastomotic tension (subjectively reported by the surgeon at the time of surgery); age at surgery; patient condition at age 1 year (alive, dead, lost to follow-up); anthropometric measures at ages 6 months and 1 year; and possible complications during the first year after birth, including anastomotic stricture, need for esophageal dilatation, TEF recurrence, gastrostomy, GERD at age 1 year, anti-reflux surgery, aortopexy, and respiratory treatment at age 1 year.

Anthropometric measures were collected by doctors during dedicated consultations. Patients were measured lying down. Length was expressed in centimeters and weight in grams.

Small for gestational age (SGA) was defined as length and/or weight Z score at birth ≤ 2 SD, according to Fenton curves (16). Delayed anastomosis was defined as anastomosis performed more than 15 days after birth, including both patients with a long gap and those with severe comorbidities that delayed surgery (i.e., cardiac malformation and prematurity).

For each patient, we calculated body mass index (BMI) Z score and length-for-age (LFA) ratio Z score at ages 6 months and 1 year using the most recent French reference growth curves (17). The curves updated in 2018 were based on an innovative big data method and are considered more representative of growth among contemporary French children (17). BMI and LFA Z scores ≤ 2 SD were defined as undernutrition and stunting, respectively. We used corrected ages at 6 months and 1 year for patients born before 41 weeks of amenorrhea (18).

Persistent GERD and the need for respiratory treatment at age 1 year were based on physician clinical evaluation.

We assessed the influences of neonatal characteristics, surgical type, and complications during the first year after birth. We compared type I EA with other EA types because the former is associated with a higher risk of surgical complications and comorbidities (19–22).

The EA register was approved by the National Informatics and Privacy Committee (Commission Nationale de l'Informatique et des Libertés) and was evaluated by the National Committee of Registers. After information was given to the parents or caregivers both verbally and in writing, all data were deidentified. Using the validated questionnaires, data were collected prospectively by specialized physicians in each tertiary care center at initial neonatal hospitalization and at 1 year follow-up. A clinical research assistant collected information from each center, and all forms were double-checked by two professionals to ensure quality and exhaustivity. The register was recorded in ClinicalTrials.gov (NCT02883725).

### Statistical analysis

Categorical variables are expressed as frequencies and percentages. Continuous variables are expressed as means (SDs), or as medians (interquartile ranges) for non-normally distributed measures. Normality of distribution was assessed graphically and with the Shapiro–Wilk test. Differences in Z scores between 6 months and 1 year were analyzed using paired Wilcoxon signed-rank tests.

Associations between baseline characteristics and undernutrition and stunting at age 1 year were performed using chi-square or Fisher exact probability tests, as appropriate. To assess independent risk factors for wasting and stunting at the age of 1 year, baseline characteristics associated with *P* < 0.20 in univariate analyses were included in a backward-stepwise multivariate logistic regression model using a removal criterion of *P* > 0.05. Results from the final model are expressed as odds ratios (ORs) and 95% confidence intervals (CIs). To avoid case deletions due to missing data, multivariate analyses were performed after handling missing values by simple imputation using a regression switching approach (chained equations with m = 1) (23). The imputation procedure was performed under the missing at random assumption using all potential factors with a binary logistic regression model.

All statistical tests were two-tailed and *P* < 0.05 was considered statistically significant. Data were analyzed using SAS software package version 9.4 (SAS Institute, Cary, NC).

## Results

### Sample characteristics

**Figure 1:**
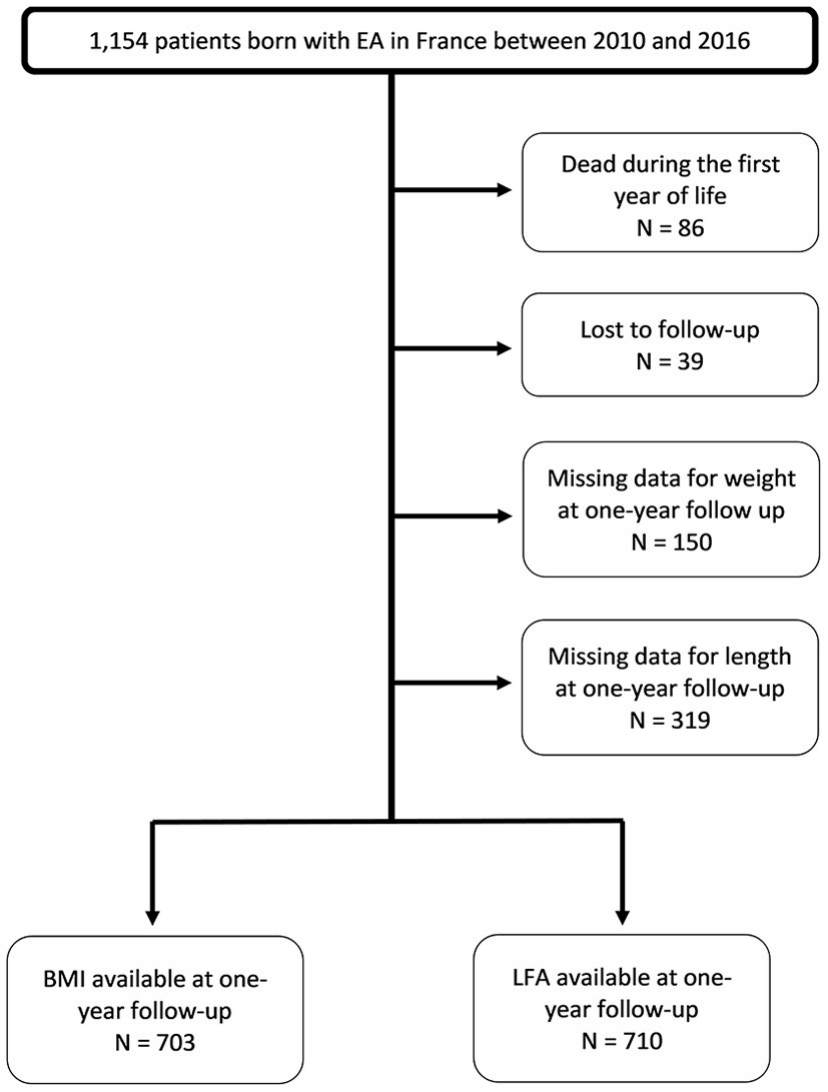
Flow chart.

We included 1,154 patients (60% male). More than 40% of the sample were born prematurely and 17% were SGA. EA was associated with TEF in over 90% of cases, with other abnormalities in 55% of cases, and as part of a syndromic association [vertebral defects, anal atresia, cardiac, TEF, renal, and limb (VACTERL) or coloboma, heart defect, atresia choanae, retarded growth and development, genital hypoplasia, and ear anomalies (CHARGE)] in 30% of cases. Esophageal anastomosis was performed in almost 95% of patients and was delayed after 15 days in 12% of cases. During the first year after birth, 86 patients (7.8%) died and 39 (3.6%) were lost to follow-up. The sample characteristics are detailed in Table 1 and Figure 1.

**Table 1:** Sample characteristics.

			**MD ^a^**
Male	*n* (%)	685 (59.4%)	0
Pregnancy	*n* (%)		0
Singleton		1099 (95.2%)	
Twins		53 (4.6%)	
Triplets		2 (0.2%)	
Prenatal diagnosis of EA	*n* (%)	287 (24.9%)	0
Weight at birth	*n* (%)	1147 (99%)	7
	mean ± SD	2,498 ± 713.1	
Length at birth	*n (%)*	865 (75%)	28
	mean ± SD	46.7 ± 4.2	9
SGA ^b^ (weight or length)	*n (%)*	118 (17%)	461
SGA for weight		159 (14.1%)	26
SGA for length		78 (9.2%)	304
Birth term (weeks of amenorrhea)	*n (%)*		23
≥37		670 (59.2%)	
32–36		364 (32.2%)	
<32		97 (8.6%)	
**Total with associated abnormality**	*n (%)*	628 (54.4%)	0
Neurologic	*n (%)*	86 (7.5%)	0
Renal	*n (%)*	113 (9.8%)	0
Cardiac	*n (%)*	326 (28.2%)	0
Limbs	*n (%)*	103 (8.9%)	0
Anorectal	*n (%)*	109 (9.4%)	0
Genital	*n (%)*	71 (6.2%)	0
Costovertebral	*n (%)*	199 (17.2%)	0
VACTERL ^c^ or CHARGE ^d^ association	*n (%)*	205 (17.8%)	0
Other syndromic association	*n (%)*	150 (13%)	0
**EA**^**e**^ **type**	*n (%)*		18
Type I		89 (7.8%)	
Type II		17 (1.5%)	
Type III		1002 (88.2%)	
Type IV		11 (1%)	
Type V		17 (1.5%)	
**Surgical treatment**			*38*
Esophageal anastomosis	*n (%)*	1090 (97.7%)	
*Age at anastomosis (days)*	mean ± SD	14.5 ± 52	*19*
Standard anastomosis	*n (%)*	1056 (94.6%)	
Anastomosis with lengthening artifice	*n (%)*	34 (3%)	
Colic transposition	*n (%)*	16 (1.4%)	
*Age at colic transposition (days)*	mean ± SD	172.3 ± 113.8	*0*
Gastric transposition	*n (%)*	10 (0.9%)	
*Age at gastric transposition (days)*	mean +/ SD	157.2 ± 69.7	*0*
Anastomotic tension	*n (%)*	323 (30.7%)	103
Timing of esophageal anastomosis	*n (%)*		19
Primary ( ≤ 15 days)		944 (88.1%)	
Delayed (> 15 days)		127 (11.9%)	
Surgical approach	*n (%)*		
Thoracotomy		960 (84.3%)	15
Thoracoscopy		143 (12.9%)	45
Cervicotomy		7 (0.6%)	27
Outcome at 1year of age	*n (%)*		64
Alive		965 (88%)	
Dead		86 (7.8%)	
Lost to follow-up		39 (3.6%)	

a *Missing data*,

b *Small for Gestational Age*,

c *Vertebral defects, Anal atresia, Cardiac, Tracheoesophageal fistula, Renal and Limb*,

d *Coloboma, Heart defect, Atresia choanae, Retarded growth and development, Genital hypoplasia, Ear anomalies*,

e *Esophageal atresia*.

### Anthropometric data

Birth weight was available for 99% of patients and birth length for 75%. Weight was available at both 6 months and 1 year for 75% of patients; length was available for 58% of patients at 6 months and 63% at 1 year. We were able to calculate Z scores at the age of 1 year for at least 60% of included patients.

Patients with missing anthropometric data at 1 year did not differ from those with available anthropometry regarding sex, SGA, birth term, associated abnormalities, prevalence of syndromic association, EA type, surgical treatment, or delayed anastomosis (Appendix 1).

Among patients with anthropometric data, 15.2% (*n* = 107/703) showed undernutrition and 19.4% (*n* = 138/710) showed stunting at 1 year. Neither BMI nor LFA Z score changed significantly between 6 months and 1 year. These data are reported in Table 2.

**Table 2:** Anthropometrics at ages 6 months and 1 year.

	**6 months**	**1 year**
**BMI** ^**a**^ **Z score**		
*n (%)*	657 (56.9%)	703 (60.9%)
Mean ± SD ^b^	−0.7 ± 2.3	−0.7 ± 2.3
Median (Q1 ^c^; Q3^d^)	−0.7 (−1.7; 0.3)	−0.6 (−1.6; 0.2)
**LFA** ^**e**^ **Z score**		
*n (%)*	662 (57.4%)	710 (61.5%)
Mean ± SD ^b^	−1 ± 1.9	−0.9 ± 1.7
Median (Q1 ^c^; Q3 ^d^)	−0.9 (−1.8; 0.1)	−0.8 (−1.8; 0)
**Undernutrition**		
BMI ^a^ Z score < −2 SD ^b^ n/*N (%)*	110/657 (16.7%)	107/703 (15.2%)
95% CI	[13.97; 19.82]	[12.57; 17.88]
**Stunting**		
LFA ^e^ Z score < −2 SD ^b^ n/*N (%)*	135/662 (20.4%)	138/710 (19.4%)
95% CI	[17.39; 23.67]	[16.53; 22.35]
**BMI** ^**a**^ **Z score delta between 6 months** ** and 1 year**		
*n*	538	
Mean	−0.01 ± 1.93	
Median	0.02	
IQR ^f^	−0.64; 0.71	
*P*	0.45	
**LFA** ^**e**^ **Z score delta between 6 months** ** and 1 year**		
*n*	546	
Mean	0.22 ± 1.67	
Median	0.01	
IQR ^f^	−0.51; 0.70	
*P*	0.11	

a *Body Mass Index*,

b *Standard Deviation*,

c *First Quartile*,

d *Third Quartile*,

e *Length-for-age*,

f *Interquartile Range*.

### Risk factors

In multivariate analyses, undernutrition and stunting were both associated with prematurity and SGA. At age 1 year, prematurity and SGA increased the risk of undernutrition by 2.43- and 2.02-fold, respectively, and the risk of stunting by 1.79- and 1.96-fold, respectively.

In addition, undernutrition was associated with VACTERL or CHARGE (OR = 2.05) whereas stunting was associated with the presence of at least one associated abnormality (OR = 1.68). These results are presented in Tables 3, 4.

**Table 3:** Predictive factors for undernutrition at age 1 year.

	**Univariate analysis**			**Multivariate analysis**		
	**Undernutrition**					
	**BMI** ^**a**^ **Z Score** < −**2 SD** ^**b**^					
	**No (*n* = 596)**	**Yes (*n* = 107)**	***P-value***	**Odds ratio**	**95% CI ^c^**	***P-value***
Sex: Male	250 (41.9%)	37 (34.6%)	0.15			
Pregnancy: Multiple (vs. singleton)	21 (3.5%)	2 (1.9%)	0.56			
Prenatal diagnosis	138 (23.2%)	35 (32.7%)	**0.04**			
SGA ^d^	97 (20.5%)	24 (28.2%)	**0.11**	2.02	(1.26; 3.25)	**0.003**
Prematurity: birth <37 weeks of amenorrhea	218 (36.6%)	61 (57%)	**<0.001**	2.43	(1.59; 3.74)	**<** **0.001**
At least one abnormality	309 (51.9%)	64 (59.8%)	0.13			
Neurologic	41 (6.9%)	8 (7.5%)	0.82			
Renal	60 (10.1%)	12 (11.2%)	0.72			
Cardiac	143 (24.0%)	29 (27.1%)	0.49			
Limbs	42 (7.0%)	13 (12.1%)	0.07			
Anorectal	50 (8.4%)	18 (16.8%)	**0.007**			
Genital	30 (5.0%)	12 (11.2%)	**0.013**			
Costovertebral	100 (16.8%)	26 (24.3%)	0.06			
VACTERL ^e^ or CHARGE ^f^ association	95 (15.9%)	31 (29.0%)	**0.001**	2.05	(1.26; 3.32)	**0.004**
Other syndromic association	65 (10.9%)	16 (15.0%)	0.23			
EA ^g^ type: Type I (vs. Types II, III, IV and V)	47 (8.0%)	10 (9.3%)	0.63			
Esophageal anastomosis (vs. colic and gastric transposition)	573 (96.1%)	105 (98.1%)	0.41			
Primary anastomosis (vs. delayed anastomosis)	537 (90.1%)	90 (84.1%)	0.07			
Thoracotomy (vs. thoracoscopy and cervicotomy)	503 (85.4%)	95 (89.6%)	0.25			

a *Body Mass Index*,

b *Standard Deviation*,

c *Confidence Interval*,

d *Small for Gestational Age*,

e *Vertebral defects, Anal atresia, Cardiac, Tracheoesophageal fistula, Renal and Limb*,

f *Coloboma, Heart defect, Atresia choanae, Retarded growth and development, Genital hypoplasia, Ear anomalies*,

g *Esophageal atresia*.

**Table 4:** Predictive factors for stunting at age 1 year.

	**Univariate analysis**			**Multivariate analysis**		
	**Stunting**					
	**LFA** ^**a**^ **Z score** < −**2 SD** ^**b**^					
	**No (*n* = 572)**	**Yes (*n* = 138)**	***P-value***	**Odds ratio**	**95% CI ^c^**	***P-value***
Sex: Male	239 (41.8%)	51 (37%)	0.30			
Pregnancy: Multiple (vs. singleton)	19 (3.3%)	4 (2.9%)	1.00			
Prenatal diagnosis	144 (25.2%)	32 (23.4%)	0.65			
SGA ^d^	87 (19.1%)	36 (33%)	**0.002**	1.96	(1.28; 3.00)	**0.002**
Prematurity: birth <37 weeks of amenorrhea	212 (37.1%)	69 (50%)	**0.005**	1.79	(1.22; 2.62)	**0.003**
At least one abnormality	288 (50.3%)	88 (64.2%)	**0.003**	1.68	(1.13; 2.48)	**0.01**
Neurologic	41 (7.2%)	8 (5.8%)	0.57			
Renal	61 (10.7%)	13 (9.4%)	0.67			
Cardiac	130 (22.7%)	42 (30.4%)	0.06			
Limbs	41 (7.2%)	14 (10.1%)	0.24			
Anorectal	52 (9.1%)	16 (11.6%)	0.37			
Genital	31 (5.4%)	11 (8%)	0.25			
Costovertebral	95 (16.6%)	32 (23.2%)	0.07			
VACTERL ^e^ or CHARGE ^f^ association	94 (16.4%)	32 (23.2%)	0.06			
Other syndromic association	59 (10.3%)	25 (18.1%)	**0.01**			
EA ^g^ type: Type I (vs. Types II, III, IV and V)	46 (8.1%)	11 (8.0%)	0.96			
Esophageal anastomosis (vs. colic and gastric transposition)	552 (96.5%)	133 (96.4%)	1.00			
Primary anastomosis (vs. delayed anastomosis)	518 (90.6%)	116 (84.1%)	0.03			
Thoracotomy (vs. thoracoscopy and cervicotomy)	488 (86.2%)	117 (86%)	**0.95**			

a *Length-for-age*,

b *Standard Deviation*,

c *Confidence Interval*,

d *Small for Gestational Age*,

e *Vertebral defects, Anal atresia, Cardiac, Tracheoesophageal fistula, Renal and Limb*,

f *Coloboma, Heart defect, Atresia choanae, Retarded growth and development, Genital hypoplasia, Ear anomalies*,

g *Esophageal atresia*.

We did not find any significant association between surgery type and any complication during the first year after birth (not presented in Tables 3, 4).

## Discussion

Despite recent advances in caring for patients born with EA, these data indicate that they remain at higher risk of undernutrition and stunting at age 1 year compared with the general population. Indeed, the prevalence of undernutrition at age 1 year during the period most recently analyzed (15.2%) is similar to that during 2008–2009 (15%) (5). The lower rate of undernutrition, compared with stunting, at 6 months and 1 year suggests harmonious growth retardation in some patients, resulting in a normal BMI. Herein, only 9.2% of patients were born SGA, whereas 20.4% were stunted at age 6 months (Tables 1, 2), suggesting that stunting at age 1 year was both constitutional and secondary to wasting.

Previous studies have reported different rates of undernutrition (8.8–20%) (6, 10) whereas, few stunting data are available (10). Our ability to compare the current findings with previous reports is limited because the latter were retrospective, based on tertiary reference centers, included small samples, and used different anthropometric markers. Lacher et al. (6) included 111 patients over a 22-year period, reporting a weight-for-age ratio below the 3rd percentile for 20% of patients at age 1 year. A recent Dutch study of 126 patients born with EA during 1999–2013 found that 8.8% had wasting and 7.2% were stunted at the age of 1 year. These lower rates can be explained by the Dutch sample's lower prevalence of prematurity (31.7 vs. 40.8% herein) and syndromic associations (12.7 vs. 17.8% herein) (10).

Another important finding herein is that undernutrition (16.7%) and stunting (20.4%) appear early, during the first 6 months after birth, though only 14% of the sample was SGA based on weight and 9% based on length. This is likely explained by these infants' associated morbidities and the complexity of their early management. No catch-up in weight or length occurred during the second half of the first year after birth, suggesting that persistent difficulties delay catch-up growth (6, 10, 24).

We found that prematurity increased the risk of undernutrition and stunting at age 1 year by almost twofold. Because preterm infants are at higher risk of being undernourished or stunted at age 1 year compared with term infants, this finding indicates that the double burden of EA and prematurity compromise nutritional status at 1 year, independent of SGA status or syndromic associations (25, 26).

Similarly, being born SGA was also strongly and independently associated with undernutrition and stunting at age 1 year, emphasizing these patients' progressive and sometimes incomplete catch-up (27, 28).

Finally, growth retardation and undernutrition were significantly and independently related to the presence of associated abnormalities, syndromic or otherwise. This suggests that associated abnormalities may play a role in stunting and wasting beyond birth anthropometrics.

These cumulative findings emphasize that undernutrition and stunting originate from early factors, determined during the fetal and neonatal period, and are independent of surgical strategy and potential complications during the first year after birth. Indeed, in contrast to previous studies, we found no significant association with GERD (7), anti-reflux surgery (10), or needing a second surgery in the first year after birth (11). Nevertheless, due to the design of our registry, objective assessment of some potential risk factors, including instrumental measurement of GERD, was lacking, which limits the strength of our conclusions.

Recent guidelines recommend the optional intervention of a dietician from age 6 months onward (29). In practice, nutritional care starts during the initial hospitalization, and growth is monitored by surgeons and pediatricians at months one and three. In view of our results, which confirm previous findings on the risks of early undernutrition and stunting, systematic early intervention by a nutritional support team should be considered. Our data highlight that particular attention must be paid to high-risk patients who are born preterm, SGA, or with associated abnormalities. Nutritional care for these patients must be closely monitored, multidisciplinary, and extended into adulthood to avoid complications related to undernutrition and to ensure optimal adult size.

This study's strengths include its uniquely large sample size, which is notable for a rare disorder like EA, thanks to the national EA register. Prospective recording of a large dataset, including prenatal information, allowed us to study a large panel of possible risk factors. One study limitation was the significant proportion of missing anthropometric data at 6 months and 1 year. Despite this, the risk of bias influencing these findings appears limited given the lack of difference between patients with or without missing data (we further reduced this risk by applying a missing data imputation process). Nevertheless, this study also presents an opportunity to reiterate the importance of repeated anthropometric measurements throughout follow-up with these patients. This study carried a low risk of selection bias because it was population-based, in contrast to most previous single-center reports.

## Conclusion

Despite consistent progress in their medical and surgical care, patients born with EA are at risk of undernutrition and stunting at age 1 year, and these impacts appear as early as 6 months after birth. High-risk patients include those born preterm, SGA, and/or with associated abnormalities; these patients may thus benefit the most from early nutritional support. Further studies are needed to monitor the long-term nutritional status at key childhood periods, into adulthood.

## Data availability statement

The raw data supporting the conclusions of this article will be made available by the authors, without undue reservation.

## Ethics statement

Ethical review and approval was not required for the study on human participants in accordance with the local legislation and institutional requirements. Written informed consent from the participants' legal guardian/next of kin was not required to participate in this study in accordance with the national legislation and the institutional requirements.

## Author contributions

SD and FG conceptualized and designed the study, collected and analyzed the data, drafted, reviewed, and revised the manuscript. AL, RS, ABo, TG, NP, P-YR, AG, TL, SI, EH, ABr, VF, HA, FE, IT, AR, MAb, J-LM, JL, PB, FS, HL, TP, YC, CB, GL, CC, CT, JB, OJ, CG, PD, MAr, CP, SG, and CL participated in data collection, reviewed and revised the manuscript. MAu and AN critically reviewed the manuscript for important intellectual content. MK carried out the statistical analyses and revised the manuscript. All authors approved the final manuscript as submitted and agree to be accountable for all aspects of the work.

## Conflict of interest

The authors declare that the research was conducted in the absence of any commercial or financial relationships that could be construed as a potential of interest.

## Publisher's note

All claims expressed in this article are solely those of the authors and do not necessarily represent those of their affiliated organizations, or those of the publisher, the editors and the reviewers. Any product that may be evaluated in this article, or claim that may be made by its manufacturer, is not guaranteed or endorsed by the publisher.

## Abbreviations

BMI: Body mass index
CHARGE: Coloboma, heart defect, atresia choanae, retarded growth and development, genital hypoplasia, ear anomalies
CI: Confidence interval
EA: Esophageal atresia
GERD: Gastroesophageal reflux disease
LFA: Length-for-age
OR: Odds ratio
SD: Standard deviation
SGA: Small for gestational age
TEF: Tracheoesophageal fistula
VACTERL: Vertebral defects, anal atresia, cardiac, tracheoesophageal fistula, renal, and limb.
