# Supplementary material for: Nutritional status at age 1 year in patients born with esophageal atresia: A population-based, prospective cohort study
Source: Front Pediatr. 2022 Aug 4;10:969617. doi: 10.3389/fped.2022.969617 (PMC9387303; doi:10.3389/fped.2022.969617)
Supplement: Supplementary file 1 [file Table_1.DOCX]

**Appendix 1**. **Comparison Between Patients With and Without Anthropometric Data at Age One Year**

|  | | Anthropometrics | | | | | |
| --- | --- | --- | --- | --- | --- | --- | --- |
|  |  | Yes  LFA ^a^ Z score  n (%) | No  LFA ^a^ Z score  n (%) | *P* | Yes  BMI ^b^ Z score  n (%) | No  BMI ^b^ Z score  n (%) | *P* |
| Male | | 427 (59.3) | 257 (59.4) | 0.99 | 419 (59.3) | 265 (59.3) | 0.98 |
| SGA ^c^ | | 122 (17.3) | 74 (17.6) | 0.89 | 118 (17) | 78 (17.9) | 0.70 |
| Prematurity | | 281 (39.7) | 180 (42.6) | 0.34 | 277 (39.9) | 184 (42.1) | 0.47 |
| Associated abnormalities | | 377 (52.4) | 251 (58.4) | 0.05 | 372 (52.8) | 256 (57.7) | 0.10 |
| VACTERL ^d^ or CHARGE ^e^ | | 125 (17.4) | 80 (18.4) | 0.64 | 124 (17.6) | 81 (18.1) | 0.82 |
| EA ^f^ type | Type I  Type II  Type III  Type IV  Type V | 56 (7.8)  12 (1.7)  626 (87.6)  7 (1)  14 (2) | 33 (7.8)  5 (1.2)  376 (89.3)  4 (1)  3 (0.7) | 0.51 | 56 (8)  12 (1.7)  613 (87.4)  6 (0.9)  14 (2) | 33 (7.6)  5 (1.1)  389 (89.4)  5 (1.1)  3 (0.7) | 0.40 |
| Esophageal anastomosis | | 696 (97.1) | 394 (98.7) | 0.075 | 682 (97) | 408 (98.8) | 0.06 |
| Primary anastomosis | | 614 (89) | 330 (86.6) | 0.25 | 600 (88.8) | 384 (87.1) | 0.42 |
| ^a^ Length-for-age, ^b^ Body Mass Index, ^c^ Small for Gestational Age, ^d^ Vertebral defects, Anal atresia, Cardiac, Tracheoesophageal fistula, Renal and Limb, ^e^ Coloboma, Heart defect, Atresia choanae, Retarded growth and development, Genital hypoplasia, Ear anomalies, ^f^ Esophageal atresia | | | | | | | |
